# Process improvement and comparability analysis for engineered T cell manufacture

**DOI:** 10.1186/2051-1426-2-S3-P29

**Published:** 2014-11-06

**Authors:** Luca Melchiori, Martin Kreutz, Daniel Williams, Gwendolyn Binder-Scholl, On Kan, David Moss, Bent Jakobsen

**Affiliations:** 1 Adaptimmune Ltd., Abingdon, Oxon, United Kingdom; 2 Adaptimmune LLC, Philadelphia, PA, USA

## 

**Figure 1:**
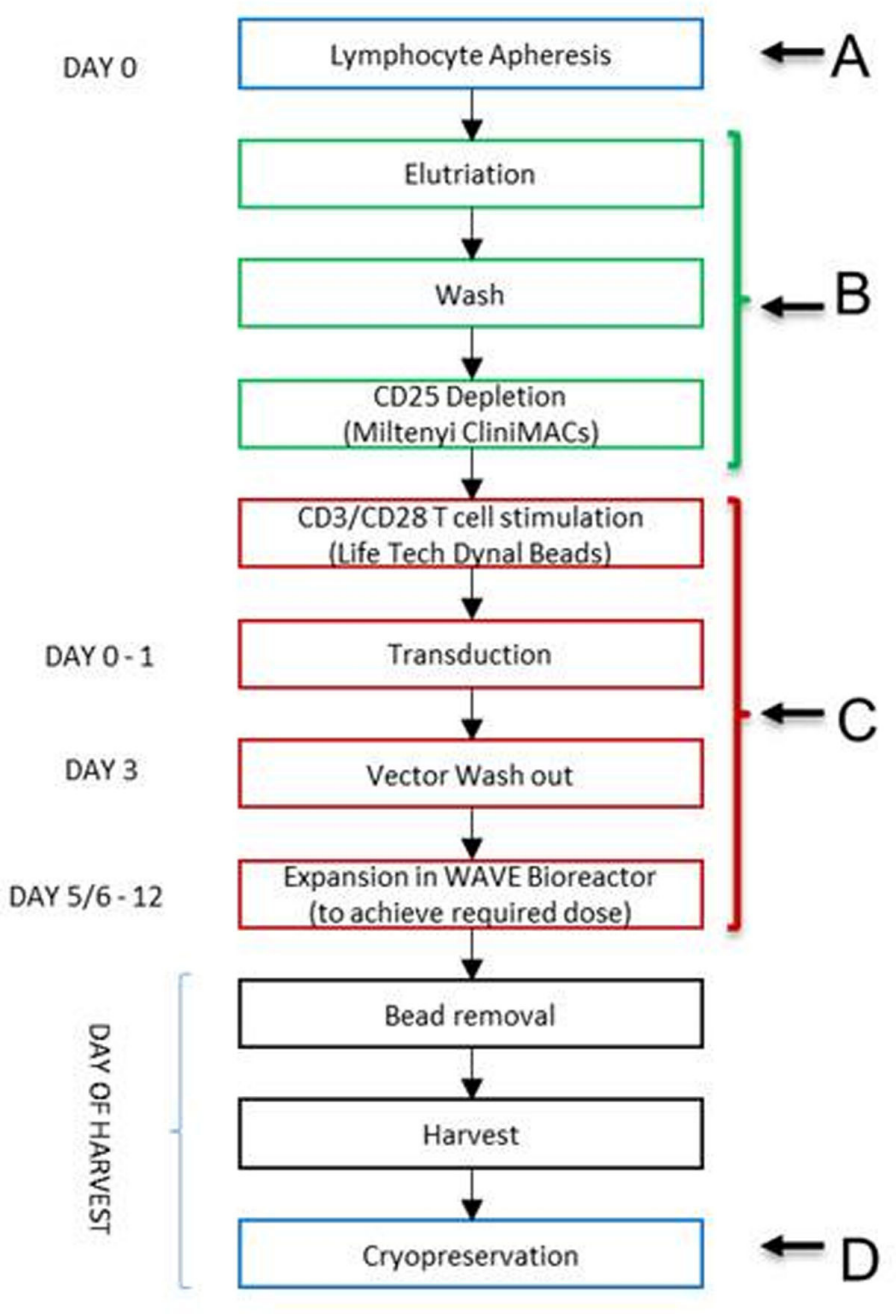
**NY-ESO-T manufacturing process**.

Engineered T cell therapy (ECT) for oncology has met significant clinical proof of success for chemotherapy resistant B cell malignancies in multiple studies. These data have underscored the transformative potential of ECT in advanced oncology. Adaptimmune specializes in the generation and testing of affinity-enhanced T cell receptors (TCR) for engineered T cell therapy, and has several ongoing clinical studies to evaluate T cells engineered with an affinity enhanced TCR specific for the NY-ESO-1 and LAGE-1 cancer testis antigens (NY-ESO-T), in patients with antigen-positive tumors. A major challenge in successful commercialization of engineered cell therapy will be to maintain product safety and potency while streamlining the manufacturing process for cost reduction, process consistency, and manufacturing portability, in order to meet commercial-scale demands and reimbursement feasibility. A summary of NY-ESO-T manufacture is provided in Figure 1. Modifications to manipulation of incoming product (A), initial T cell enrichment (B), medium components (C), and final formulation (D) will be described. To evaluate safety and efficacy of the product after these process changes, comparability studies have been carried out, including 14-colour flow cytometry analysis of cell phenotype and functionality after expansion. Data will cover the phenotype of T cell sub-populations, cytokine expression profile, exhaustion and activation marker expression, to demonstrate product consistency and potency following process changes. Establishment of critical quality attributes will require iterative analysis of correlative and efficacy data from clinical trials.

